# Electricity system security in Jordan: A response for arab uprising

**DOI:** 10.1016/j.heliyon.2023.e15961

**Published:** 2023-05-04

**Authors:** Ahmad Alshwawra, Ahmad Almuhtady, Ahmad Sakhrieh

**Affiliations:** a Leibniz University Hannover, Hannover, Germany; b Mechanical and Maintenance Engineering Dept., School of Applied Technical Sciences, German Jordanian University, Amman-Madaba Street, P.O. Box 35247, Amman 11180 Jordan; c Department of Mechanical and Industrial Engineering, School of Engineering, American University of Ras Al Khaimah, United Arab Emirates; d Department of Mechanical Engineering, School of Engineering, The University of Jordan, Amman, Jordan

**Keywords:** Electricity system security, Energy policy, Arab uprising, Energy security, Jordan, Renewables, Energy security indices, Energy geopolitics, Energy politics

## Abstract

Jordan energy sector is characterized by the high dependency on imported energy and high growth rate of energy demands. The location of Jordan in a conflict hot spot makes the energy security of high interest for the Jordanian policy maker. This article investigates the impact of regional conflicts on Jordanian energy sector and tracks the development of electricity system security before and after the first wave of Arab uprising and the turmoil associated with it. An electricity sector security framework consisting of eleven indices is built based on Stirling four properties of energy security, which are durability, stability, robustness and resilience. The framework is used to compare the security of the system in 2018 with 2010. This article argues that the development in the security during the study period is a response of the Arab uprising based on authoritarian learning phenomena. The results are validated by comparing the expected generation costs and CO_2_ emissions based on actual development with development scenarios found in literature. A forecasting model is reproduced for this purpose. The results of the forecasting model support the conclusion reached by the security framework. This is due to the responsive policies followed by the Jordanian government and the grants given by Gulf countries to enhance Jordan’s stability. It was concluded that even a specific conflict can have negative impacts on the energy sector in a neighboring country in short terms, it can have positive impacts in medium and long term if a rational and sustainable response plan is adopted.

## 1 Introduction

The 1973 Arab oil embargo gave energy a political dimension, since it grew the fear of the usage of oil as a political weapon [1]. In 2014, the G7 energy ministers had met in Rome to discuss how to “disarm Russia’s energy weapon” [2]. The recent Russian – Ukrainian war shows the economic impacts of energy weapon. It was admitted that in the multi-polar world we live in, and with the anarchic nature that characterizes the international system, developing an efficient, rational and adaptive energy sector is a challenge for each state [3]. This led to the securitization of the energy; i.e. governments frame energy as an existential threat to state interests [4].

The concept of energy security got a lot of attention in the last two decades. This can be seen by the significant increase in the related publications [5]. However, the energy security concept is still highly contested. According to Ang et al. [6] literature contains more than Eighty different definitions for energy security. Most of them focused on energy availability, infrastructure, and social effects aspects. In the last ten years, environmental, governmental and efficiency-related aspects are getting more attention in energy security studies. The International Energy Agency’s (IEA) definition for the energy security focuses on the continuity of the supply and prices’ stability. IEA also connected the definition of energy insecurity to the welfare loss resulting from energy interruption or high prices [7].

The relationship between conflict and energy security had been discussed by many researchers [[8], [9], [10], [11]]. The impact of a certain conflict can exceed the disputants’ energy systems, and overspill to other neighboring countries, or even have International implications [12,13]. Conflicts implications on the energy system in neighboring countries include increasing in energy demands; primarily due to the refugee waves, interruption in the energy flow due to impacting the well-being of the energy supply chain, or increasing the energy costs [14,15]. Many evidences show that conflicts; directly or indirectly; have resulted in energy exporters altering their energy prices worldwide [16].

Jordan lies in the Middle East, However; it lacks substantial fossil fuel reserves [17]. Whenever the Jordanian energy sector topic is raised, four important remarks must be taken into consideration. Firstly, Jordan is characterized as a heavily energy importer as it is largely dependent on fossil fuels imports to meet the local energy demands. The Jordanian local energy resources have a very small contribution in the total energy mix [18]. Secondly, the energy demands in Jordan are characterized of being of high growth rate that exceeded 10% in some years [19]. It should be emphasized that this high growth rate has not been met by an equivalent economic growth [20]. Thirdly, Jordan’s geographic proximity to internationally renowned fossil fuels exporters gives Jordan a reasonable access to energy sources. Lastly, Jordan is located in a conflict hot spot. This increases the risk of being an energy importer [21].

On the one hand, some regional conflicts threatened the Jordanian energy security. For example, the insurgencies associated with the Lebanese civil war in 1975 led to unfavorable change in the operation characteristics of the TAPLINE; the single oil source in Jordan in that period. Moreover, the associated refugee wave increased the Jordanian energy demands [14,21]. Furthermore, the first Gulf war in 1990 witnessed the use of the TAPLINE as a political weapon against Jordan [22]. Nonetheless, it was the 2003 invasion of Iraq which represented one of the most impacting events by stripping Jordan from its only energy provider at that time and consequently, creating catastrophic ramifications on the Jordanian economy [21]. On the other hand, some conflicts actually ended up enhancing the Jordanian energy security, such as the Iraq-Iran war in the 1980s, which had increased the economic integration with Iraq and established Iraq a secured source for cheap energy [10,14].

In the beginning of 2011, the Arab world experienced a sudden sequence of demonstrations and revolutions, known as the Arab uprising. The Arab uprising brought down many regimes, and in some cases resulted in a state of lawlessness and civil wars in some of the Arab countries. While the Jordanian regime was not directly affected in the Arab uprising, indirect consequences of the chaos in the neighboring countries affected Jordan on political, economic and social levels [23,24]. Energy is one of the sectors that were impacted by the turmoil, especially in Egypt, Syria and Iraq [25]. The Jordanian government applied a series of policy adaptations to absorb the implications of those conflicts, mainly in the electricity sector. Additionally, hundreds of millions of dollars were granted by the Gulf countries, which have been invested in the energy sector to enhance Jordan’s stability. This led to major changes in Jordan’s energy security within the period 2010–2018.

Azzuni and Bayer [5] measured the energy security in Jordan quantitively using a comprehensive energy security index. Even with the scarce energy resources, they ranked Jordan at 118 of the world energy security level, which is about within the middle of the standing. This was mainly due to Jordan’s high performance in health and military dimensions, and reasonable performance in policy and technology. However, they did not provide a track of how the Jordanian energy security changed over years and the reasons of these changes. Another study examined the energy security for 100% renewable energy transition in Jordan in 2050 [26]. It concluded that the transition for 100% renewables will enhance the energy security level in Jordan by improving the energy availability, energy cost, environment, health, and employment in Jordan.

Electricity represents the most ready to use form of energy, which have propelled its share of the total energy consumption (on country level) starting from the last century and going forward [27]. Even traditionally known fossil-fuel-reliant applications such as transportation and heating are progressively migrating towards electricity; prompted by higher efficiency operations and lower CO2 emissions [[28], [29], [30]]. Moreover, while a large share of electricity is still being generated from fossil fuel (especially in smaller countries such as Jordan), renewable energy electricity generation has been gaining more momentum in both large and small countries. The latter means that at some point in the future, electricity security frameworks will be de-attached from fossil fuel aspects. Finally, in terms of interruptions, electricity is the most directly felt energy form in residential and industrial applications [31,32]. Therefore, the security of the electrical sector is naturally one of the most important energy securities, if not the most.

This study targets a thorough investigation of the direct impact of the conflicts associated with the first wave of Arab uprising on the Jordanian energy sector, and to evaluate the development in the electricity sector in Jordan within the period between 2010 and 2018 to determine if it had led to a more secure electricity system or have undermined it. To do so, an event study method is used in the beginning to study the short term impacts of the conflicts. After that, desk research is utilized to develop an electricity sector security framework. Then a comparative analysis assessing the situations in 2010 and in 2018 is held using the developed security framework. Lastly, the results of the comparative analysis are validated using a forecasting model found in literature. The validation is done by comparing the expected generation costs and CO_2_ emissions based on the actual developments with the scenarios that exists in literature.

The novelty of this study can be summarized in the following: Firstly, it presents the short and medium terms impacts of the regional conflicts associated with the first wave of Arab uprising on the Jordanian electricity sector. Secondly, it analyzes the results of the response plan and augmented policies adopted by the Jordanian government with respect to the electricity sector. Thirdly, it presents an interpretation of the reasoning behind the changes in the Jordanian electrical sector based on the authoritarian learning phenomena.

The article structure is laid as follows: In the next section a brief description of the electricity sector in Jordan will be presented. Then the implications of the conflicts in the period of study on the Jordanian energy sector will be discussed. After that, the electricity system security framework will be built, and its indicators will be described and applied for the study period. Consequently, the results will be discussed and lastly, the forecasting model will be presented and re-simulated to validate the results.

## 2 Jordanian electricity system

**Fig. 1:**
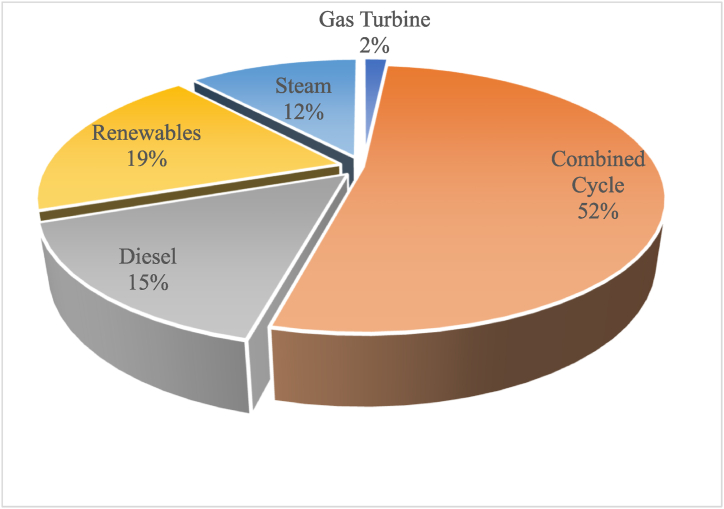
Installed Generation Capacities at the end of 2018. Data Source [34].

**Fig. 2:**
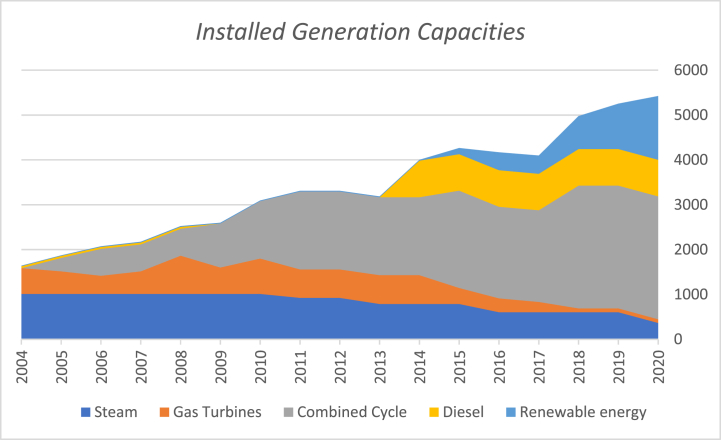
Installed generation capacities 2004–2018. Data source [34].

Al-Omary et al. [33] presented a sufficient description for the organizational structure of the electrical sector in Jordan. However, the Generation sector was updated by the emergence of more entities and companies belonging to the private sector, especially in the field of renewables [19]. The total generation capacity at the end of 2018 was 5236 MW including 994 MW of renewable energy. Fig. 1 shows the installed generation capacities in Jordan at the end of 2018. The renewable energy generation capacities include 698.5 MW solar, 280.4 MW wind, 3.5 MW biogas, and 12 MW hydropower [34]. Fig. 2 shows the installed generations capacities for the period 2004–2020. Fig. 2 reflects the changes the electricity generation sector witnessed over the mentioned period. There was a steady increase in combined-cycle generation capacity while a decrease in gas turbine generation capacity. A significant part of Jordan's electricity generation capacity has been generated by diesel since 2013. During the past few years, Jordan started relying on renewable energy to generate electricity.

**Fig. 3:**
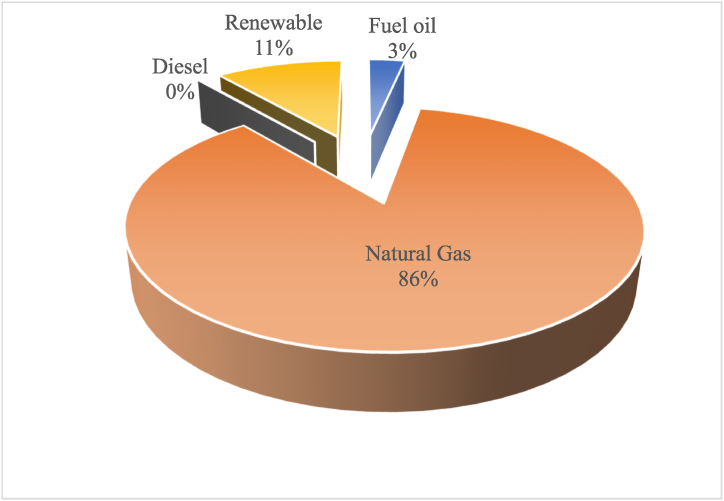
Fuel share in the total electricity generated in 2018. Data source [34].

**Fig. 4:**
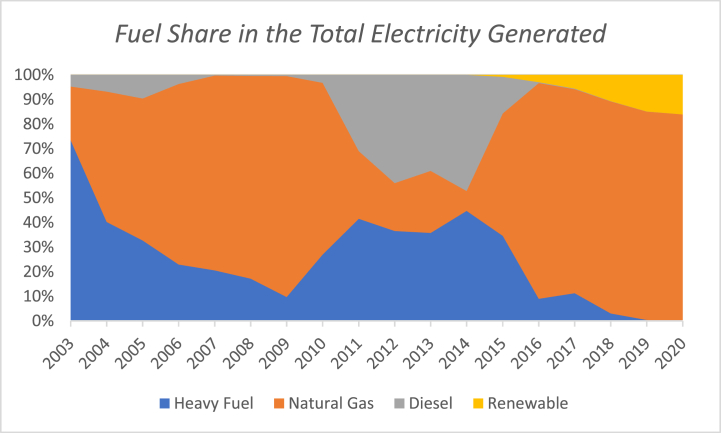
Fuel share in the total electricity generated 2004–2018. Data source [34].

Diesel installed generation capacity can be operated by different types of fuel (Natural gas, fuel oil, and Diesel). This increases the resilience of the electricity system to absorb any sudden interruption of supply. The above power plants generated 20,502 GWh in 2018 [34]. The share of each fuel in the total generated electricity is shown in Fig. 3. As it could be seen from the figure, natural gas is the dominant fuel in the electricity sector at this year, reclaiming its number-one-place after it had lost dominancy in the period 2011–2015 (see Fig. 4). The latter was caused by the interruption in the supply from Egypt, following a state of lawlessness post the Egyptian revolution [21]. Regaining the dominancy happened due to the opening of the Jordanian Gas Port (The Shaikh Sabah Al Ahmad Al Sabah Port) in Aqaba in 2015.

The percentage of electricity generated by different fuels over the period 2004 to 2020 is shown in Fig. 4. Since 2004, Jordan has been using natural gas in place of heavy fuel till 2010. As a result of the difficulties associated with securing natural gas for the period from 2010 till 2015, Jordan had to go back and rely on heavy fuel and diesel for electricity generation. In the period after that and starting in 2015, Jordan was able to once again to rely on natural gas for electricity generation increasing its share and reducing the share of heavy fuel. It is also worthy to mention that since 2014 renewable energy's share has increased. A comparison between 2010 and 2018 shows that in both years Jordan relied on natural gas to generate more than 80% of the electricity required to satisfy its demands. However, a more-in-depth look reveals that the case is really not the same. In 2010, Jordan had relied solely on Egypt as a source of its NG imports via the Arab Gas Pipeline, which was the case since 2004 [19]. In 2018, Jordan diversified the NG sources through receiving NG shipments through the Gas Port in Aqaba. This diversity of suppliers enhances the security of the electricity sector. Moreover, the incorporation of renewable energy resources available in 2018 increased the durability and robustness of the system; future prospect wise.

**Fig. 5:**
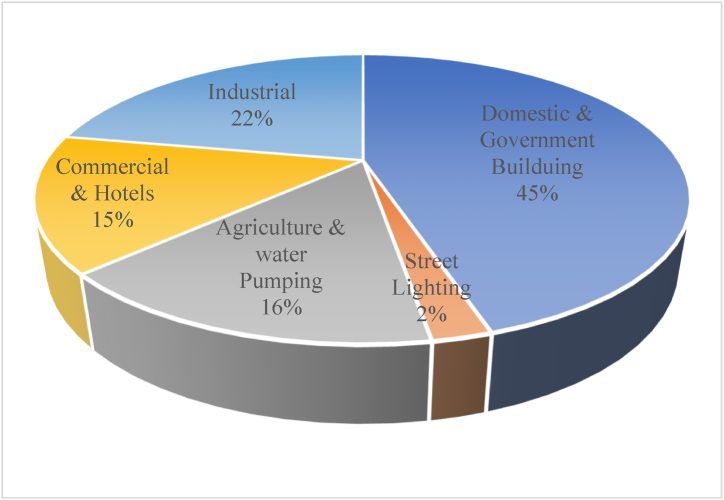
Electricity Consumption in 2018 by sector. Data Source [34].

The consumed electrical energy in Jordan in 2018 was 17,494 GWh [34]. Fig. 5 shows the distribution of the consumed electricity by sector. It could be seen in Fig. 5 that the residential and commercial sectors jointly grasp the majority of the electricity consumption in Jordan (about 60%). Those sectors are too sensitive to climate conditions, energy prices (fossil fuels and electricity), economic growth, cultural and religious habits, and population growth [35]. Moreover, this indicates that the electricity sector in Jordan is highly susceptible to the refugee waves, compared for example to an industrial country where a larger share of electricity would be used for production and manufacturing.

## 3 Conflicts implications

There are many conflicts that has sparked in the MENA region between 2010 and 2018. These have affected Jordan in more than one level and one dimension. Some of these conflicts had adverse impacts on the Jordanian energy sector resulting from an increase in the demand because of refugees’ waves or due to interruption of supply of oil and gas. For the former, the turmoil in Syria, Iraq and Yemen are examples of that. Whereas the Egyptian revolution and the insurgencies in Iraq are examples for the latter. Those conflicts motivated the government of Jordan to change its Energy policies and accelerate the application of its energy strategic plan [36].

**Fig. 6:**
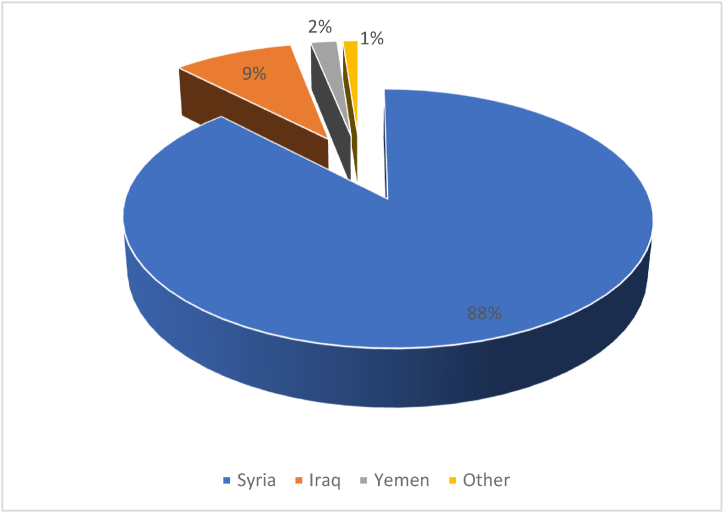
Refugees’ distribution in Jordan according to their countries of origin. Source [37].

Jordan received a huge number of refugees in the study period. According to UNHCR, Jordan hosts refugees from 57 countries [37]. More than 90% of those refugees were received between 2010 and 2018. Fig. 6 shows the refugees distribution according to their country of origin. As it can be seen from the figure, the majority of them are form neighboring Syria due to the civil war which erupted in 2011. Yemen refugees also started to flee to Jordan after 2011. On contrast, most of registered Iraqi refugees were in Jordan before 2010 [38]. It is worthy to mention that the number of registered refugees can be as low as half of the total actual number. For example, according to UNHCR there are about 700,000 registered Syrian refugees in Jordan, while according to the Jordanian official statistics there are about 1.4 million Syrians living in Jordan [39].

**Fig. 7:**
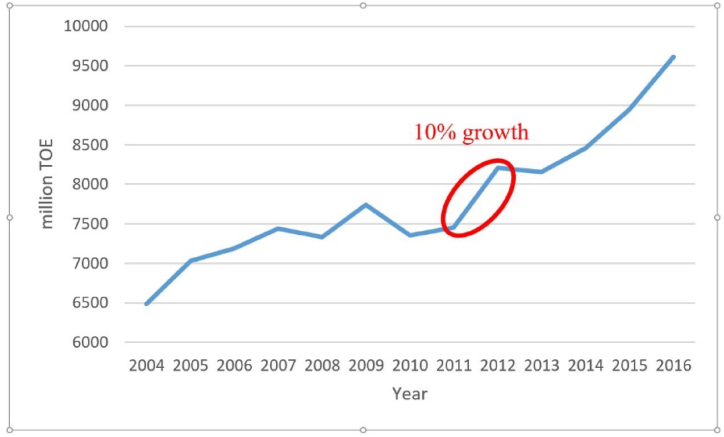
Growth of primary energy demands. Data source [19].

Since more than 80% of those refugees live outside the refugees’ camps, i.e. in the Jordanian cities, this instigated a huge load on the infrastructure of those cities [37]. Energy demands were affected sharply; mainly due to Syrian refugees. The Ministry of Energy and Mineral Resources (MEMR) statistics demonstrate that the primary energy demands jumped by 36% from 7457 million tons of oil equivalent (TOE) in 2010–10,009 million TOE in 2017 [19]. The highest growth rate was 10% occurring in 2012 when Jordan received the highest number of Syrian refugees. This rate is almost twice the energy demands growth in the period 2004–2010, as can be seen in Fig. 7. The energy demands has grown by 13% from 6489 TOE in 2004–7457 TOE in 2010 [19] The increase of the energy demand growth rate from an average of 2.2% per year in the period 2004–2010 to 5.1% per year in the period 2010–2017 is a clear evidence of the refugees impact on the Jordanian energy sector.

**Fig. 8:**
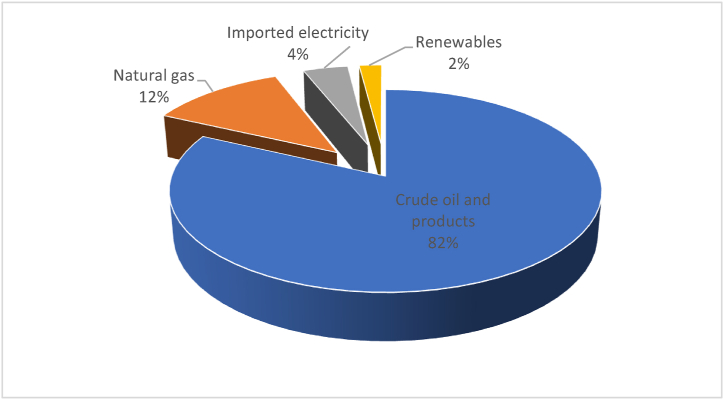
Primary energy mix in 2010. Data source [19].

Crude oil and natural gas were dominating the Jordanian energy primary mix in 2010 (Fig. 8). Both of them were susceptible to the neighboring conflicts. Starting in 2008 and on a daily basis, tens of road tankers had been getting through the Jordanian eastern borders carrying Iraqi Oil to Jordan. Jordan used to import 10,000 barrels of oil per day from Beji - Kirkuk in Iraq [19,36]. This amount had represented about 10% of the daily Jordan needs of crude oil before 2010. Iraq had sold this oil to Jordan with a favorable price that was $18 cheaper than the global prices at the time [40]. After, deducting the transportation costs of $13 per barrel, Jordan used to save $5 per barrel from those quantities. These savings were interrupted in 2014, since Jordan was unable to import oil from Iraq due to the complete loss of security on the shipment roads with increased insurgencies, and later the rise of ISIS [36].

Jordan started to import the Egyptian natural gas (NG) through the Arab Gas pipeline in 2004. The Egyptian gas represented 12% of the Jordanian primary energy mix as shown in Fig. 8. The imported gas was used to generate 80% of the electricity in Jordan [19]. Jordan had even promising plans of increasing the imported Egyptian gas, which is to be followed by the establishment of a NG domestic piping network infrastructure in Jordan’s biggest cities; Amman and Zarqa in order to provide NG to the industrial, commercial, and residential sectors [19]. However, the 2011 Egyptian revolution had brought down Mubarak’s regime, and the Arab Gas pipeline became an obvious target of sabotage, being bombed more than ten times; because it was supplying both Jordan and Israel, with the latter’s policies against Palestinians. At that time, Jordan was left without its only gas supplier. The ramifications on the Jordanian electricity sector were catastrophic. In the beginning of 2012, Qutaiba Abu Qura, the former Jordanian Minister of Energy, issued a statement claimed that Jordan was losing 4 million US$ daily as a result [41].

## 4 A framework for the security of electricity system

Despite the country’s energy security being the most sought-after target in energy policies, in this study more focus will be put on the factors that affect the security of the electricity system. This can be justified by two facts. The first is that the electricity system is one of the most vital energy security subsystems; if not the most. This can be deuced by noting that more than 40% of Jordan’s yearly primary fuel consumption is attributed to electricity generation [34]. The second is that the electricity sector was the most affected sector due to the neighboring conflicts in the period of study. The proposed framework will be drawn from the widely accredited works of Andy Stirling that recognizes four properties for security, namely; stability, durability, resilience, and robustness [42].

Diversifying energy sources (suppliers) and resources (fuels) are the center of any security framework [6,[42], [43], [44], [45], [46]]. That is the generation sector’s stability and durability can be attained from such diversity, not to mention achieving resilience and robustness. The diversity of resources can be measured by counting the fuels used for electricity generation. The diversity of sources will be measured by counting the gas suppliers, since the majority of electricity in Jordan is generated using natural gas. Increasing the domestic resources contribution in electricity generation will favorably enhance the stability, durability, and robustness of the electricity system [14,21,35,43,47,48]. Nonetheless, the system’s durability and robustness cannot be achieved without increasing the share of renewable energy resources in the electricity generation [6,46,[49], [50], [51]]. This share is measured by the renewable energy resources contribution in the installed generation capacities.

An efficient forecasting for electricity demands on yearly, monthly, daily, and even hourly basis is very important for the stability and the durability of the system. This forecasting must take into consideration the impact of multiple effects such as temperature, fuel prices, economic growth, etc. [35,43,46,49,52]. The indicator for this index is the performance of the forecasting by the responsible company; NEPCO; and the announcement of these forecasting results. The durability and stability of the system also instigates the safeguarding of the electricity supply chain, and achieving a state of cooperation and integration with other countries; where the latter can be done for example via interconnection transmission lines [43,49]. As domestic sabotage to the electricity system is minimal, the protection will be measured by the number of vulnerable supply chain elements concerning the primary fuel used for generation (NG). The integration will be measured by the number of interconnected countries.

To achieve a significant resilience for the system, optimal fuel safety margins have to be employed to guarantee the crucially needed buffer against shocks and paves the road for reasonable recovery after disruptions [6,43,44,49]. Inherently, one direct way to measure it would be identifying the period (in days) the strategic storage is able to meet the country’s energy demands in case of fuel(s) interruption. The resilience also requires a sufficient spare parts stock, mainly for the critical parts needed for electric power production and distribution [6,43,44,49]. As the actual information of the electricity system spare parts stocks is unavailable, an indirect indicator can be attained by measuring the average duration of interruptions in the transmission stage. Moreover, if the same power plant has the flexibility of utilizing more types of fuel, that can only positively impact the resilience too [46]. The measurement of this index can be achieved by counting the number of different fuels the primary electricity generation can work with.

**Fig. 9:**
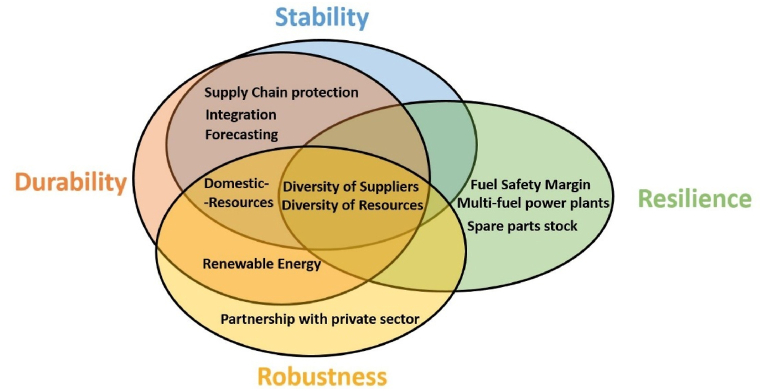
Proposed framework for electricity system security.

Lastly, partnership with the private sector and joint ventures in the electricity sector will further promote the system’s robustness; where competition usually elevates the levels of efficiency in the energy market [43,44,49]. Identifying the number of the companies and entities belonging to the private sector which are involved in the generation and distribution stages can represent the measure here. The suggested framework is illustrated in Fig. 9. It can be seen that there is sufficient number of indices employed in this framework (eleven) as Ang et al. [6] had stated that more than half of the published energy security frameworks (in the period 2001–2014) employed eleven indices or less. Consequently, one can deduce that the number of indices used in the current framework is sufficient to measure the electrical system’s state of security in Jordan.

## 5 Results and discussion

Utilizing the aforementioned framework, results of the electricity system security in Jordan, relevant to the variation that took place between 2010 and 2018, are displayed in Table 1. In 2010, Jordan imported natural gas from Egypt, while in 2018 Jordan was open to the International gas market. This happened due to the commissioning of the Gas port in Aqaba in the end of 2015. The number of fuels (energy sources) used for electricity generation in 2010 was 3; Gas, diesel and heavy fuel [34]. That number increased to 5 in 2018, due to incorporating renewable energy sources, such as solar energy and wind energy, in the generation stage as has been depicted in Fig. 2. The domestic fuel share in the electricity generation increased from 0% in 2010 to 10.7% in 2018, due to the newly installed renewables generation capacities. These renewables contribution in the total installed generation capacity has increased from 0% in 2010 to 19% the in 2018 [34].

**Table 1:** Results for electricity system security in 2010 and in 2016. Data Source [19,34].

Index	Indicator	2010	2018
**Diversity of Suppliers**	No. of Gas suppliers	1	Gas Market
**Diversity of Resources**	No. of fuels used in elec. Generation	3	5
**Domestic Resources**	Domestic fuel share in elec. Generation	%0	%10.7
**Renewable Energy**	% of total installed generation capacity	%0	%19
**Forecasting**	Forecasting performed and announced	Yes	Yes
**Integration**	No. of Interconnected countries	4	8
**Supply Chain protection**	Fuel supply chain in unprotected areas	1	0
**Partnership with private sector**	No. of private sector involved	7	46
**Fuel Safety Margin**	Adequacy of strategic storage	14 days	60 days (2019)
**Multi-fuel power plants**	No. of fuels that can be used	3	3
**Spare parts stock**	Duration of interruptions in transmission stage	40 min/intr.	30 min/intr.

Both in 2010 and 2018, NEPCO performed the electricity demands forecasting and announced it [34]. By 2010, Jordan had established interconnection with Egypt, Syria, and Libya. By 2018, new countries have joined the interconnection namely; Palestine, Lebanon, Turkey, and Sudan [34]. In 2010, a major part of the gas pipeline transporting NG to Jordan was located in Sinai Peninsula, which suffered insecurities with lack of control by the Egyptian government. This pipe was an indispensable part for the gas supply chain for Jordan. However, the situation changed in 2018 after opening the LNG port in 2015 as mentioned above. The partnership with the private sector was significantly increased in 2018 due to the emergence of new renewable energy companies in the generation market. The number in 2018 was 46 [19].

Jordan introduced new strategic storage capacities in the south and center of Jordan to increase the sufficiency of the strategic storage from 14 days demands to 60 days demands. The power plants in Jordan have been always capable of operating with Gas, Diesel and heavy fuel. Thus there is no change on this index between 2010 and 2018. The average duration of interruption in the transmission stage fell from 40 min/interruption in 2010 to 30 min/interruption in 2018 [34]. Based on these results, one can conclude that the security of the electricity system in Jordan has been significantly improved in 2018 compared with 2010.

This improvement can be attributed to the response of Jordan governments to the consequences of the Arab uprising. Jordan secured 5 billion USD as grants from the Gulf countries in order to support the stability in Jordan [53]. Those billions were used to build the LNG terminal in Aqaba, the strategic gas storage in Aqaba, the strategic storage for oil and gas in Amman, and to promote the renewable energy projects. Basically, these funds were the backbone for the upgrade of the security of the electricity system in Jordan. It helped Jordan enhance the diversity of sources and resources, increasing the shares of domestic resources and renewables, increase the fuel safety margin, and pave the road for more cooperation with the private sector.

This generous fund can be explained by the phenomena of Authoritarian learning, which is “a process in which authoritarian regimes adopt survival strategies based upon the prior success and failures of other government” [54]. Monarchies in the Arab world survived the Arab uprising, since they were able to distance themselves from the failure of their governments’ policies by firing them [55,56]. The Gulf countries witnessed the transfer of regime falling from one republican country to another; mainly because of economic situation, but no monarchy was dethroned. Nonetheless, some studies at the time raised the red flag of the criticality of the Jordanian economy, and its potential adverse impacts on the stability of the Jordanian monarchy [[56], [57], [58]]. Therefore, the Gulf monarchies were motivated to positively step in and rectify the economic situation through generous grants to avoid a Domino Effect scenario. After all, the primary concern of any authoritarian regime is to stay in power [59], hence the rich gulf countries supported Jordan’s stability by just-in-time grants [56]. Based on that, it could be said that the bolstered-up security in Jordan’s energy sector in general, and its electrical sector in specific was a response for the Arab uprising.

## 6 Framework’s results validation

To validate the conclusion reached by the results of the framework, the expected electricity generation costs and the expected accompanying CO_2_ emissions in 2030 based on the projection of actual development, will be compared with the costs and emissions based on development scenarios found in literature. The forecasting model developed by Al-omary et al. [33] will be used to make this comparison. Al-omary et al. predicted that according to Business As Usual (BAU) growth rate, a generation capacity of 5790 MW is required in 2030. Expecting that the existing generation capacity will decline to 2724 MW in 2030; they predicted that an extra 3076 MW of generation capacity is needed [33]. They also introduced three development scenarios for electricity generation system from now up towards 2030. Those three scenarios were; (1)100% generation capacity from fossil fuels, (2) 84% from fossil fuels and 16% from solar, and (3) 84% from fossil fuels and 16% from wind. Those three scenarios were built without considering the development in electricity sector after 2013, despite the model being published recently.

The forecasting model was reproduced in order to predict the generation costs and the amount of CO_2_ emissions considering the actual developments. All the current electricity generation projects that have been implemented since 2013 and the ones under implementation or in the tendering stage will be taken into consideration. This includes renewable energy projects, oil shale power plant projects, and power plant rehabilitation projects. In order to benchmark the results of this scenario with the results of the proposed scenarios in literature, same assumptions and values will be considered.

Based on the demand prediction in BAU scenario and the actual project development, Jordan in 2030 will need 2000 MW of additional generation capacity besides the current generation capacity. This capacity will be selected as 4 × 500 MW IGCC power plants to be similar to the other scenarios. The model also includes a 500 MW gas turbine, working with minimum capacity, as a backup system for wind and PV systems. This gas turbine has been as well included in the other scenarios. Based on that, Table 2 shows the expected capacity in 2030, classified by the type of operating fuel.

**Table 2:** The expected generation capacities in 2030 based on the projection of actual development.

Fuel	Capacity	%
Oil shale	470 MW	5.8
Wind turbine	700 MW	8.6
PV system	1200 MW	14.7
IGCC	4483 MW	54.9
Gas Turbine	500 MW	6.0
Diesel/HFO/LNG	814 MW	10.0
**Total**	**8167 MW**	**100**

**Fig. 10:**
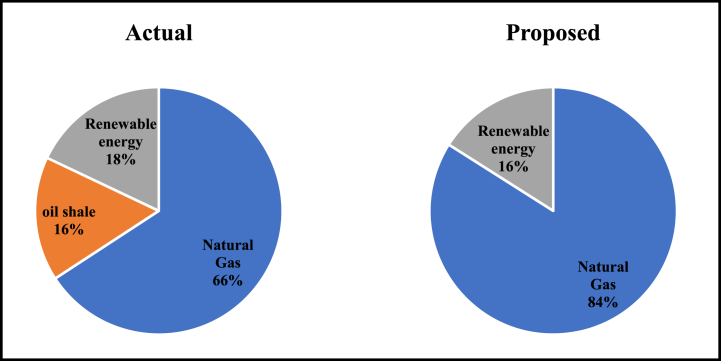
The expected fuel share in electricity generation in 2030.

Fig. 10 shows the expected fuel mix in electricity generation in 2030 for the scenario built on actual development in this article, and the scenario suggested by Al-omary et al., (2018). It can be seen that the expected renewable energy share in electricity generation based on the scenario built on actual data is higher. This means that the government of Jordan have done a great Job in exploiting renewable energy. This conclusion agrees with literature [60]. Moreover, the existing of the oil shale shows that Jordan is in the way of utilizing its domestic natural resources. Jordan has a huge reserve of oil shale that could be effectively utilized for electricity generation through direct burning [61]. Currently there is a project of 470 MW oil shale power plant under implementation at the center of Jordan.

All prices and technical assumptions of the reference article will be used to calculate the cost of kWh. The cost for oil shale power plant is not considered in the reference article. Hence, it will be assumed to be similar to that one of pulverized coal ($3700/kW or 2627 JD/kW) with operational and maintenance cost at (35 US$/kW or 24.9 JD/kW) [62]. Jordanian oil shale has a LHV of 7000 kJ/kg [63]. This assumption is justified since direct burning technology will be utilized to produce electricity from Jordanian oil shale. Therefore, 38% efficiency power plant will produce 737 kWh/ton. Regarding CO_2_ emissions, 0.819 kg CO_2_/kWh emissions is produced when generating electricity using oil shale. Finally, the cost of oil shale is assumed 20 JD/ton.

The predicted generation price for kWh in 2030 was calculated using Equation (1):

(1) $$c_{a,P}=I_{p}\frac{i{\left(1+i\right)}^{L}}{{\left(1+i\right)}^{L}-1}+F_{P}+O\&M_{p}$$

Where $c_{a,P}$ is the total yearly cost for any generation technology P, $I_{P}$ is the overall capital cost for technology P. L is the lifetime of the respective power plant P, which is considered 25 years. i is the yearly interest rate which is considered to be 4%. $F_{p}$ is the yearly fuel cost for technology P if any, and $O\&M_{p}$ are the yearly costs for operation and maintenance.

**Fig. 11:**
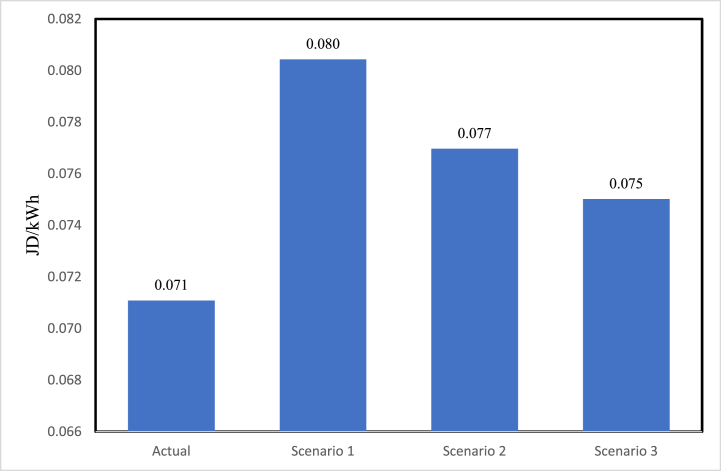
The expected cost for electricity generation in 2030.

**Fig. 12:**
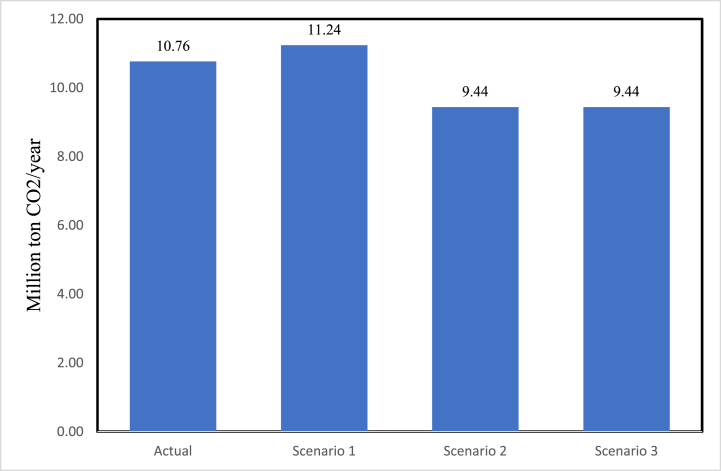
The expected amount of CO_2_ emitted in electricity generation in 2030.

Fig. 11 shows the expected cost for electricity generation in 2030. It could be seen that the kWh price for the scenario built on the actual development is the lowest. The proposed scenario of 84% fossil fuel and 16% wind energy (scenario 3) is lower than scenario 2 (84% fossil fuel and 16% PV) and scenario 1 (100% fossil fuel). Fig. 12 presents the expected amount of CO_2_ emitted in electricity generation in 2030. The scenario built on the actual development is expected to emit more CO_2_ than scenarios 2 and 3. This is due to the incorporation of oil shale in the generation mix. However, the amount of emitted CO_2_ in this scenario is lower than the 100% fossil fuel scenario (scenario 1).

The results above comply with the results concluded using the proposed electricity system security framework. Both show that the actual development in the electricity system in Jordan has been enhancing its security. This will be beneficial for reducing the electricity generation cost. The selling cost to the end consumer is affected by the taxation and subsidies policies followed by the government, as up till now, the government is the sole decision maker in regards to the final electricity prices in Jordan [64]. Moreover, Jordan needs to give more attention to the environmental aspects since its plans of utilizing the huge oil shale reserves are ongoing. Environmentally-friendly technologies, as well as technologies which tackle the reduction of the fossil fuel burning emissions can be adopted to avoid increasing the amount of CO_2_ (and other pollutants) emissions.

## 7 Conclusion

Jordan is a heavy energy importer with high energy demands growth rate that is located in a conflict hotspot. The energy sector in Jordan suffered many times over the past 70 years due to the regional conflicts. This makes energy security an issue of great interest for policy makers. Starting from 2011, the Jordanian energy sector was impacted by the turmoil associated with the Arab uprising. The Jordanian government applied a series of policy changings, in addition to investments of hundreds of millions of dollars from grants in the energy sector which promoted Jordan’s energy security. This article investigated the development in the most important energy sector in Jordan; namely the electricity sector; between 2010 and 2018 to track the electricity system security evolution in this period.

During the study period, the energy sector in Jordan was adversely affected by the regional conflicts in two ways; refugee waves and generation fuel interruptions. Jordan received hundreds of thousands of refugees from Syria, Iraq, and Yemen. This increased the demands significantly, especially in 2012 when the energy demands jumped by 10% from the previous year. The electricity sector in Jordan is too susceptible to the refugee waves as the residential and commercial sectors dominate (with 60%) the electricity consumption in Jordan. On the other hand, Jordan imported about 10% of its daily oil demands from Iraq with a preferable price. This was interrupted in 2014 due to the insurgencies in Iraq. Furthermore, Jordan lost the NG supply from Egypt due to the chaos following the Egyptian revolution. The ramifications of that on the electricity generation sector were substantial.

The electricity system security framework was developed based on Stirling four properties of security, which are: stability, durability, resilience, and robustness. It consists of eleven indices: diversity of generation fuel suppliers, diversity of fuels used in generation, the contribution of domestic resources, the contribution of renewable energy resources, sufficient forecasting, supply chain protection, electrical system integration, partnership with private sector, fuel safety margin, multi-fuel power plants and spare parts stock. Half of the published energy security framework between 2001 and 2014 used 11 indices or less, henceforth this framework is sufficiently elaborate to measure the change in security. The results of this framework shows that electricity system security in Jordan increased significantly in 2018 compared to 2010.

The framework results were validated using a forecasting model found in the literature. The expected electricity generation cost and the expected CO_2_ emissions in 2030 based on the projection of actual development, were compared with the cost and emissions based on development scenarios found in literature. It was found that the expected generation cost based on the actual development is less than the ones based on the scenarios suggested in literature. However, the CO_2_ emission needs to be given careful consideration due to the incorporation of the domestically available as a natural resource oil shale in the generation fuel mix. This agrees with the conclusion reached using the security framework.

It can be concluded from this case study, that even a specific conflict can have negative spill over impacts on the energy sector in a neighboring country in short terms, it can have positive impacts in medium and long term, especially, if rational energy policies are adopted and more investments in sustainable energy resources are established. This could be applicable to the current Russian – Ukrainian conflicts where the negative impacts were spilled over to the energy sector in many European countries. In such cases, adopting a rational and sustainable response plan would be more beneficial in medium and long term.

## Funding statement

The publication of this article was funded by the Open Access Fund of the 10.13039/501100001664Leibniz Universität Hannover.

## Author contribution statement

Ahmad Alshwawra: Conceived and designed the experiments; Performed the experiments; Analyzed and interpreted the data; Contributed reagents, materials, analysis tools or data; Wrote the paper.

Ahmad Almuhtady, Ahmad Sakhrieh: Analyzed and interpreted the data; Contributed reagents, materials, analysis tools or data; Wrote the paper.

## Data availability statement

Data included in article/supp. material/referenced in article.

## Declaration of competing interest

The authors declare that they have no known competing financial interests or personal relationships that could have appeared to influence the work reported in this paper.
